# Transcriptome analysis of rice root responses to potassium deficiency

**DOI:** 10.1186/1471-2229-12-161

**Published:** 2012-09-10

**Authors:** Tian-Li Ma, Wei-Hua Wu, Yi Wang

**Affiliations:** 1 State Key Laboratory of Plant Physiology and Biochemistry (SKLPPB), National Plant Gene Research Centre (Beijing), College of Biological Sciences, China Agricultural University, #2 West Yuan Ming Yuan Rd, Beijing, 100193, China

**Keywords:** Rice, K^+^ deficiency, microarray, transcriptome

## Abstract

**Background:**

Potassium (K^+^) is an important nutrient ion in plant cells and plays crucial roles in many plant physiological and developmental processes. In the natural environment, K^+^ deficiency is a common abiotic stress that inhibits plant growth and reduces crop productivity. Several microarray studies have been conducted on genome-wide gene expression profiles of rice during its responses to various stresses. However, little is known about the transcriptional changes in rice genes under low-K^+^ conditions.

**Results:**

We analyzed the transcriptomic profiles of rice roots in response to low-K^+^ stress. The roots of rice seedlings with or without low-K^+^ treatment were harvested after 6 h, and 3 and 5 d, and used for microarray analysis. The microarray data showed that many genes (2,896) were up-regulated or down-regulated more than 1.2-fold during low-K^+^ treatment. GO analysis indicated that the genes showing transcriptional changes were mainly in the following categories: metabolic process, membrane, cation binding, kinase activity, transport, and so on. We conducted a comparative analysis of transcriptomic changes between *Arabidopsis* and rice under low-K^+^ stress. Generally, the genes showing changes in transcription in rice and *Arabidopsis* in response to low-K^+^ stress displayed similar GO distribution patterns. However, there were more genes related to stress responses and development in *Arabidopsis* than in rice. Many auxin-related genes responded to K^+^ deficiency in rice, whereas jasmonic acid-related enzymes may play more important roles in K^+^ nutrient signaling in *Arabidopsis*.

**Conclusions:**

According to the microarray data, fewer rice genes showed transcriptional changes in response to K^+^ deficiency than to phosphorus (P) or nitrogen (N) deficiency. Thus, transcriptional regulation is probably more important in responses to low-P and -N stress than to low-K^+^ stress. However, many genes in some categories (protein kinase and ion transporter families) were markedly up-regulated, suggesting that they play important roles during K^+^ deficiency. Comparative analysis of transcriptomic changes between *Arabidopsis* and rice showed that monocots and dicots share many similar mechanisms in response to K^+^ deficiency, despite some differences. Further research is required to clarify the differences in transcriptional regulation between monocots and dicots.

## Background

Potassium (K^+^) is the most abundant monovalent cation in plant cells and accounts for 2–10% of plant dry weight [1]. It plays crucial roles in many physiological processes in living plant cells, including osmoregulation, control of turgor pressure, electrical neutralization, and enzyme activation [1,2]. The K^+^ concentration in soil solution is generally within the range of 1 to 200 ppm (approx. 0.025 ~ 5 mM) [3]. In the rhizosphere, the K^+^ concentration is usually less than 0.3 mM [4]. Therefore, most plants are subjected to low K^+^ stress at some point during their lives. Most plants can survive under low-K^+^ conditions, largely because of the high-affinity K^+^ uptake system in the roots, especially the high-affinity K^+^ transporters [5]. Different plant species and genotypes have various high-affinity K^+^ transporters with diverse K^+^-uptake efficiencies [6].

Previous studies reported that many high-affinity K^+^ transporter genes are induced by low-K^+^ conditions. For example, several members of the *KUP/HAK/KT* family including *AtKUP3*[7], *AtHAK5*[8,9], *HvHAK1*[10,11], *OsHAK1*[12] as well the K^+^ transporter genes *TaAKT1*[13] and *AtCHX17*[14] were induced by K^+^ starvation. These K^+^ transporters may be involved in K^+^ acquisition and homeostasis in plant cells under low-K^+^ conditions. Furthermore, transcriptomic analysis of *Arabidopsis* under low-K^+^ stress identified many candidate genes related to low-K^+^ perception, and regulatory pathways associated with responses to K^+^ deficiency [9,15].

Microarray technology is a convenient tool for rapid analysis of plant gene expression patterns under a variety of environmental conditions. Research on signal transduction pathways activated during the plant response to nutrient deficiency is often initiated from microarray data analyses [16]. For instance, the genome-wide gene expression profiles of rice responses to drought [17], low nitrogen [18], low phosphorus [19-21] and low iron [22] stresses have been analyzed using microarray techniques. However, very little progress has been made in understanding changes in transcription of rice genes under low K^+^ conditions.

In the present study, we used Affymetrix microarrays to monitor the transcriptomic profiles of rice roots during responses to short- and long-term K^+^ deficiency. We analyzed the functional categorization of differentially expressed genes and compared the low-K^+^ response profiles between rice and *Arabidopsis*. These results will further our understanding of the molecular mechanisms underlying responses to nutrient deficiency in plants.

## Results and discussion

### K^+^ deficiency inhibits growth of rice seedlings

To investigate the transcriptional changes in rice roots under K^+^ deficiency, we first determined the appropriate period of low-K^+^ treatment. Hydroponically grown rice seedlings (2-weeks old) were transferred to K^+^-free solution (−K) and K^+^-replete solution (+K, 1 mM K^+^) as treatment (LK) and control (CK) conditions, respectively. The nutrient solution used was that described by the International Rice Research Institute [23]. After treatment for indicated times (6 h, and 1, 3, 5, and 7 d), the K^+^-deficient phenotypes of rice seedlings were evaluated. The dry weight and K^+^ content were also measured at each time point.

**Figure 1:**
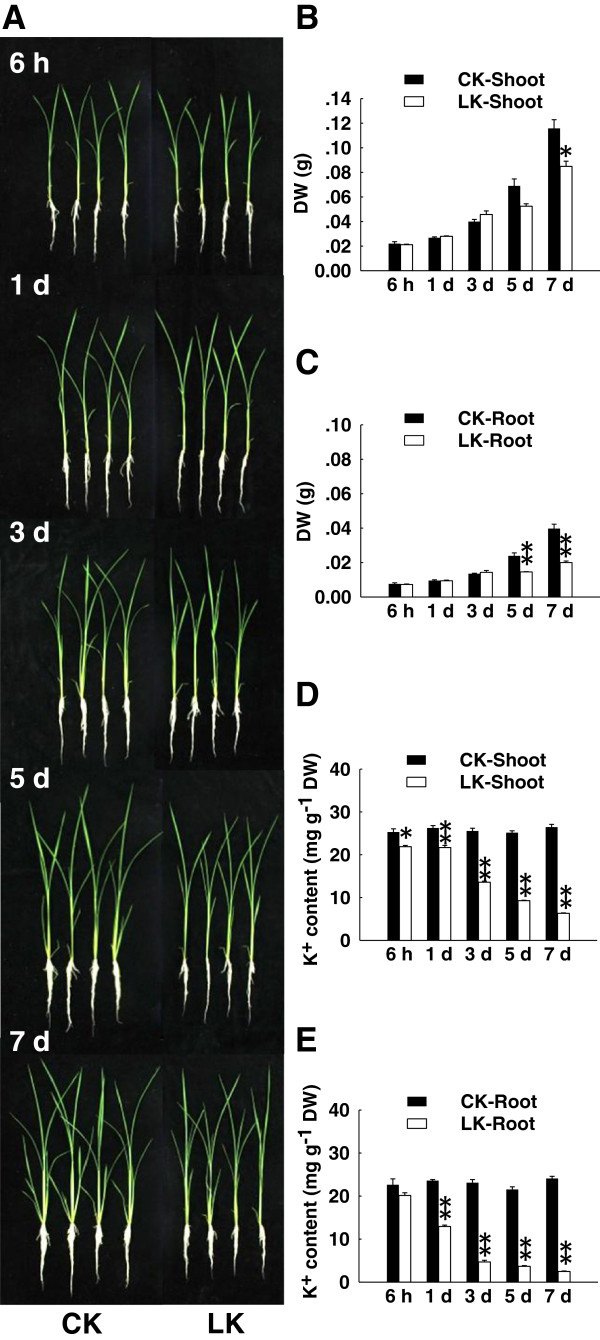
**K**^**+ **^**deficiency inhibits rice growth and affects seedling K**^**+**^**content.** (**A**) Phenotypes of rice seedlings under normal and K^+^-deficient conditions (CK and LK) at indicated times. (**B**, **C**) Dry weight of shoots (**B**) and roots (**C**) under CK and LK conditions at indicated times. (**D**, **E**) K^+^ content of shoots (**D**) and roots (**E**) at indicated times. Data are means ± SEM (*n* = 3). Differences between mean values of treatments and controls were compared using *t* - tests (* *P* < 0.05, ** *P* < 0.01).

There was no phenotypic difference between K^+^-starved seedlings and control seedlings after K^+^ deficiency treatment for 6 h, or 1 or 3 d (Figure 1A). The seedlings showed obvious phenotypic differences after 5 days of K^+^ starvation, at which time the K^+^-deficient plants had smaller roots and shoots than those of the control (Figure 1A). The observed phenotypes were consistent with the biomass measurements. Compared with control seedlings, the biomass of roots and shoots of K^+^-starved seedlings was lower after 5 d K^+^ deficiency (Figure 1B, 1C). The seedling K^+^ content showed a decrease after only 6 h, and continued to decrease markedly throughout the K^+^ deficiency treatment (Figure 1D, E).

According to these results, we chose three time points (6 h, and 3 and 5 d) to investigate changes in gene transcription. The seedling K^+^ content had already decreased at the 6-h time point. It decreased further thereafter, but biomass was not affected until the 3-d time point. At the 5-d time point, the K^+^ content and biomass were both significantly decreased. Therefore, these three time points may represent different stages of rice root responses to K^+^ deficiency.

### Microarray analysis reveals rice genes showing transcriptional changes in the response to K^+^ deficiency

**Figure 2:**
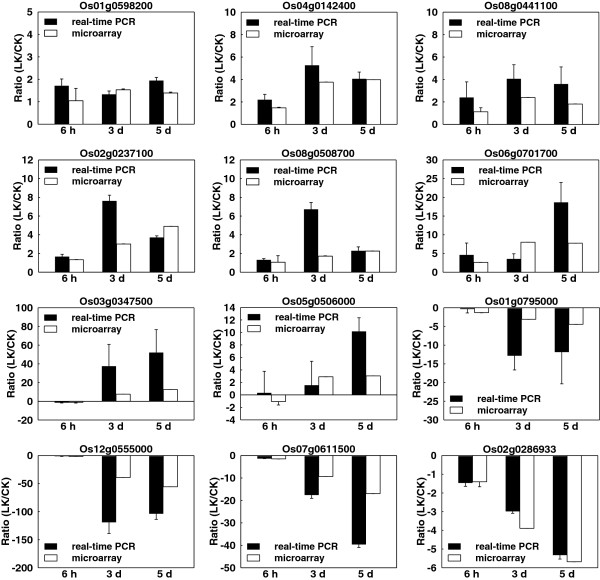
**Confirmation of transcriptomic profiles of selected genes by real-time PCR.** LK/CK ratios in microarray data of 12 tested genes compared with real-time PCR results. Sequences of gene-specific primers used for PCR are listed in Additional file 12.

We used an Affymetrix rice genome array to reveal transcriptional changes during the rice response to K^+^ deficiency. This array contains 51,279 probes representing two rice cultivars (48,564 *japonica* transcripts and 1,260 *indica* transcripts, http://www.affymetrix.com). The root is the main tissue that first senses nutrient deficiency in the environment. Therefore, roots from K^+^-starved seedlings and controls were harvested at the three time points (6 h, 3 d, and 5 d). Their total RNA was extracted and used for microarray experiments (see “Materials and Methods” for details). To ensure reproducibility and reliability of microarray data, three biological replicates were analyzed at each time point, making a total of 18 rice genome arrays. To assess the reproducibility of microarray data, the correlation coefficients of biological replicates were calculated using GeneSpring GX 11 software. All correlation coefficients were greater than 0.9915 (Additional file 1). We conducted real-time PCR analysis for 12 randomly selected genes to confirm the validity of the microarray data. For most of the 12 genes, their expression patterns in the real-time PCR analysis were similar to those predicted by the rice microarray data (Figure 2). The raw data sets (CEL) and the normalized expression data sets have been deposited in the Gene Expression Omnibus (GSE37161) at the National Center for Biotechnology Information [24] (http://www.ncbi.nlm.nih.gov/geo/).

The microarray results showed that approximately 25,000 genes showed positive signals in this data set. This represented approximately 44% of the probe sets on the rice genome array (Additional file 2). Among these expressed genes, we were more interested in those that showed transcriptional changes in response to low-K^+^ conditions. We analyzed these differentially expressed genes using DNA-Chip Analyzer (dChip) 2007 software [25] (http://www.dchip.org). This revealed genes whose expression levels in K^+^-starved seedlings were up-regulated or down-regulated more than 1.2-, 1.5-, 2.0-fold (*P*-value < 0.05) compared with control seedlings (see “Materials and Methods” for details). In total, 2,896, 1,166, and 356 genes showed changes in expression of more than 1.2-, 1.5-, and 2.0-fold, respectively (Additional file 3). Since there were fewer up- or down-regulated genes at the 1.5 or 2.0-fold cutoff thresholds, we used the 1.2-fold cutoff threshold for further analysis in order to contain more candidate genes involving in rice response to K^+^ deficiency.

The number genes showing changes in expression increased as the K^+^-starvation time increased from 6 h to 3 d to 5 d (Table 1). At the beginning of the K^+^-starvation period (6 h and 3 d), there were more up-regulated genes (100 at 6 h, 719 at 3 d) than down-regulated genes (64 at 6 h, 486 at 3 d) (Table 1). This result suggested that many K^+^-related genes were induced, and that these may be involved in the response and adaption to K^+^-deficiency during this period. However, at a later stage of K^+^-starvation (5 d), there were more down-regulated genes (1186) than up-regulated genes (1141) (Table 1). This indicated that the expression of many genes may have been restrained to slow the growth rate of rice, while others were activated to adapt to long-term K^+^-deficiency.

**Table 1:** **Numbers of differentially expressed genes (DEGs) in rice during K **^**+ **^**-deficiency. Up-regulated genes (Up) and down-regulated Genes (Down) at different LK-treatment times are shown **

**Gene Category**	**No. of Genes**	**Percentage of total DEGs**
Up-6 h	100	3.5
Up-3 d	719	24.8
Up-5 d	1,141	39.4
Down-6 h	64	2.2
Down-3 d	486	16.8
Down-5 d	1,186	41.0

Previous transcriptomic studies in *Arabidopsis* showed that only a few genes were transcriptionally regulated by K^+^ deficiency [9,15]. In contrast, phosphorus (P) and nitrogen (N) deprivation caused transcriptional changes in a vast number of genes in *Arabidopsis*[26,27]. We observed similar trends in rice seedlings. There were fewer genes showing transcriptional changes in the present study than in previous studies on rice seedlings subjected to P- and N-starvation [18-21]. These findings, together with previous data, indicate that post-translational regulation may be more important than transcriptional regulation in plant responses to low-K^+^ stress [9,16].

**Figure 3:**
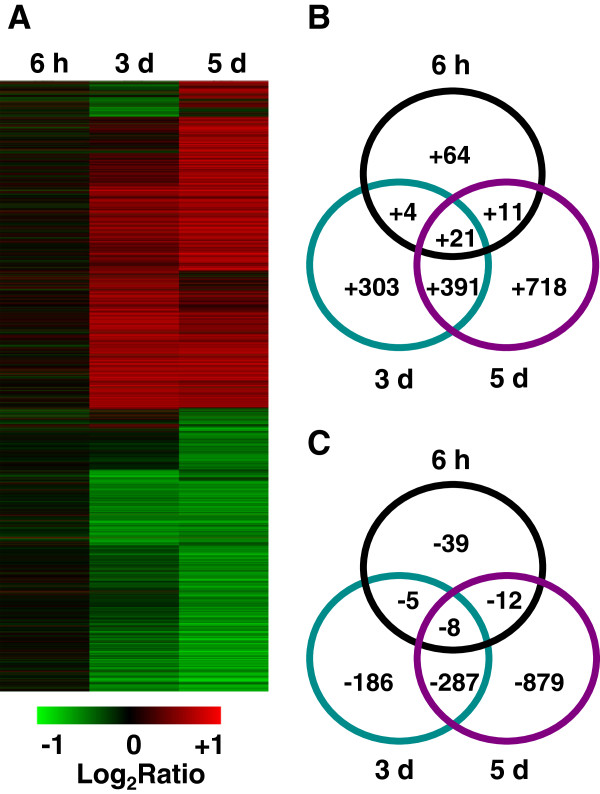
**Hierarchical cluster analysis and Venn diagrams of rice genes showing transcriptional changes in response to K**^**+ **^**deficiency.** (**A**) Hierarchical cluster analysis of genes showing transcriptional changes at indicated times (6 h, 3 d and 5 d) during K^+^ deficiency. (**B**, **C**) Venn diagrams of up- (**B**) and down- (**C**) regulated genes at different LK-treated times. The genes up-regulated at least at one time point were selected for the analysis in (**B**). Meanwhile, the genes down-regulated at least at one time point were selected for the analysis in (**C**).

To investigate the similarities and differences among genes showing changes in transcription at different time points during the low-K^+^ treatment, we carried out a hierarchical cluster analysis and constructed a Venn diagram (Figure 3). The genes showing changes in expression at 3 and 5 d showed similar expression patterns, which were different from those of genes showing changes in expression at 6 h (Figure 3A). This suggested that the genes showing transcriptional changes at 6 h may represent those involved in short-term responses to K^+^ deficiency, whereas those involved in long-term responses may begin to be activated 3 days later. In addition, some genes showed transcriptional changes at all time points measured—these may have functions throughout the entire period of K^+^-starvation (Figure 3B, C).

### Gene ontology analysis of differentially expressed genes in the rice response to K^+^ deficiency

**Figure 4:**
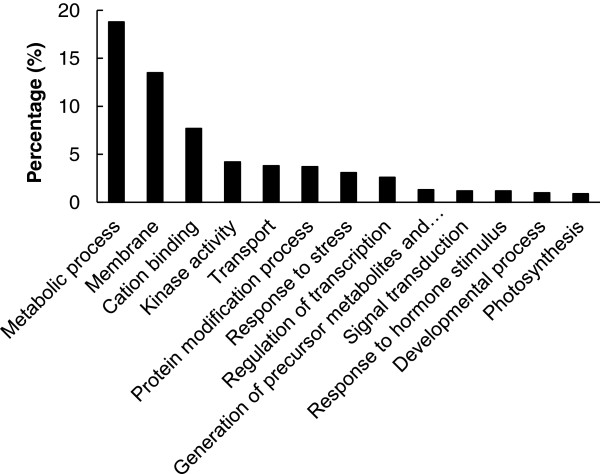
**Functional category distribution of rice genes showing transcriptional changes in response to K**^**+**^**deficiency.** AgriGO web-based tool was used to analyze GO categories of genes showing changes in transcription levels. Percentages of genes showing transcriptional changes in 13 main biological categories are shown.

To evaluate the potential functions of these differentially expressed genes in the response to K^+^ deficiency, Gene Ontology (GO) analysis was performed using the AgriGO online service [28] (http://bioinfo.cau.edu.cn/agriGO). The 2,896 differentially expressed genes were classified into 13 functional categories including metabolic process (18.8%), membrane (13.5%), cation binding (7.7%), kinase activity (4.2%), transport (3.8%), protein modification (3.7%), and response to stress (3.1%), as shown in Figure 4. Most of these processes were considered to be closely related to the response to K^+^-deficiency. Further analysis showed that in most of the GO categories, there were far more up-regulated than down-regulated genes at the 6-h and 3-d time points (Additional file 4), but more down-regulated than up-regulated genes at the 5-d time point (Additional file 4). The results of GO analysis may provide some clues to understand the transcriptomic profiles of genes involved in rice responses to K^+^ deficiency.

### Metabolic enzymes

**Figure 5:**
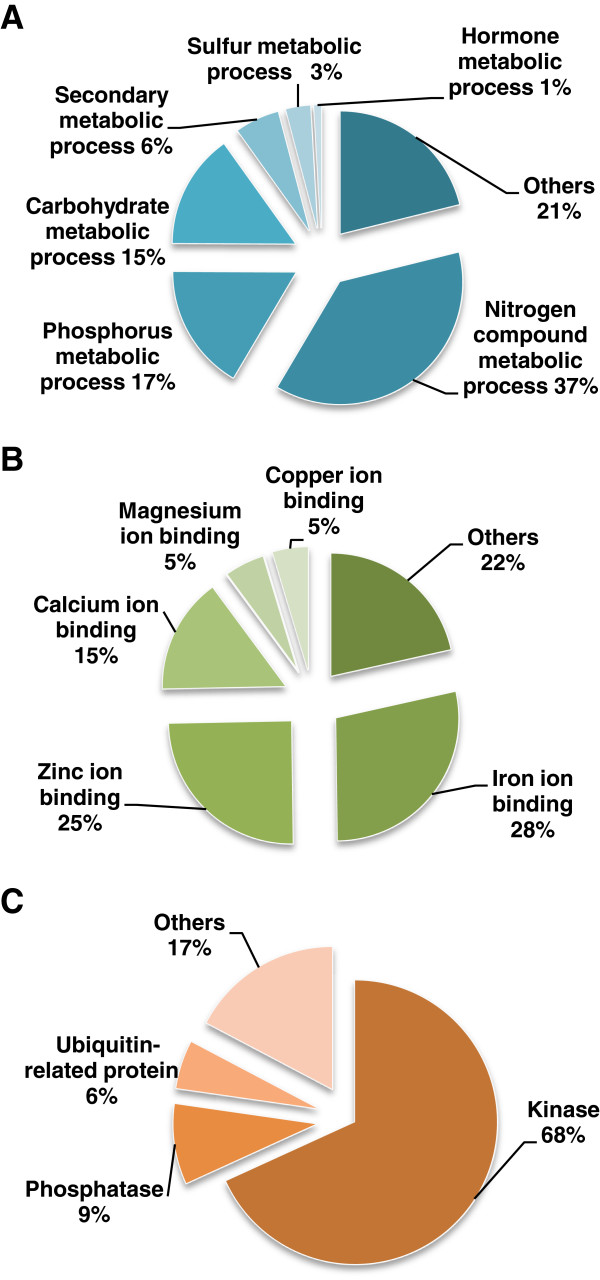
**Functional classification of genes showing transcriptional changes in three main categories.** Detailed classification of genes showing transcriptional changes in the categories of metabolism process (**A**), cation binding (**B**), and protein modification process (**C**) were analyzed using AgriGO web service.

Further analysis of differentially expressed genes in the metabolic process category indicated that approximately 37% were related to nitrogen metabolism, 17% to phosphorus metabolism, and 15% to carbohydrate metabolism, together accounting for approximately 70% of genes in this category (Figure 5A). Many metabolic enzymes require K^+^ ions as a cofactor [29]; therefore, a decrease in cytosolic K^+^ concentration may influence metabolic processes. A previous study reported that transcriptional regulation of metabolic enzymes may be very important in adaptation to K^+^ deficiency in *Arabidopsis*[30].

In this study, the expression levels of two nitrate reductase (*NR*) genes (Os08g0468100 and Os08g0468700) decreased at a late stage (5 d) of K^+^ deficiency. Some protein kinases and phospholipases that play roles in phosphorus-related metabolic processes were also transcriptionally regulated. Two *PEPCK* (phosphoenolpyruvate carboxykinase) genes (Os10g0204400 and Os01g0208700) were significantly down-regulated at the 5 d time point.

Many metabolites in *Arabidopsis* roots, such as pyruvate, malic acid, and nitrate, significantly decreased when plants were subjected to low-K^+^ stress [30]. However, 24 h after resupply of K^+^ in the growth medium, the concentrations of most of these metabolites had recovered [30]. Changes in the cellular concentrations of metabolites may result from K^+^-induced transcriptional changes in genes encoding metabolic enzymes. Consequently, changes in the K^+^ supply level would affect their transcription levels. Taken together, these results suggested that long-term K^+^ deficiency may affect the regulation of metabolic processes, leading to depressed cellular activities and a slower plant growth rate to enable the plant to survive in nutrient-limited conditions.

### Cation binding proteins

The differentially expressed genes in the cation binding category mainly encoded proteins that bind iron, zinc, and calcium ions (Figure 5B). We found that approximately one-third of the iron-binding protein genes encoded peroxidases (POXs), which are heme-containing proteins in plant cells [31]. POXs play crucial roles in scavenging reactive oxygen species (ROS) during the processes of cellular metabolism and abiotic stresses [32,33]. Previous research indicated that accumulation of ROS in plant roots is an early response to K^+^ deficiency [34,35], and that many peroxidase genes related to ROS signaling are induced under low-K^+^ conditions [15,34]. In the present data, 21 peroxidase genes showed changes in transcription under low-K^+^ conditions in rice (8 up-regulated and 13 down-regulated). This confirmed the relationship between peroxidases and low-K^+^ signal sensing in rice. These transcriptional regulated peroxidase genes are classified into six subgroups [36], and listed in Additional file 5.

We also identified nine calcium sensor protein genes showing changes in expression levels during K^+^ starvation; five were up-regulated (*OsCBL5*, *OsCML1*, *OsCML18*, *OsCML20*, *OsCML31*) and four were down-regulated (*OsCML5*, *OsCML11*, *OsCPK9, OsCCaMK1*) (Additional file 5). Previously, Ca^2+^-binding proteins were reported as being important K^+^-responsive genes in *Arabidopsis*[15]. Here, we also identified Ca^2+^ sensor proteins in rice, which indicated these proteins may connect Ca^2+^ signaling and down-stream target proteins in the plant response to K^+^ deficiency.

### Kinases and protein modification

In the protein modification category, most genes were identified as kinases (68%) and phosphatases (9%) (Figure 5C). This result suggested that phosphorylation and dephosphorylation may be very important regulatory mechanisms in the rice responses to K^+^ deficiency. Previous investigations showed that many protein kinases modulated ion transport processes in living cells by regulating the activity of the corresponding ion transporters under ionic stress. This regulatory mechanism is found in both plant and mammalian cells [37-39]. The results of GO analysis in the present study showed that many genes in the kinase and protein modification categories were transcriptionally regulated by K^+^ starvation (Figure 4). The kinases encoded by these genes may be involved in regulating K^+^ uptake and K^+^ homeostasis in rice under K^+^ deficiency.

**Figure 6:**
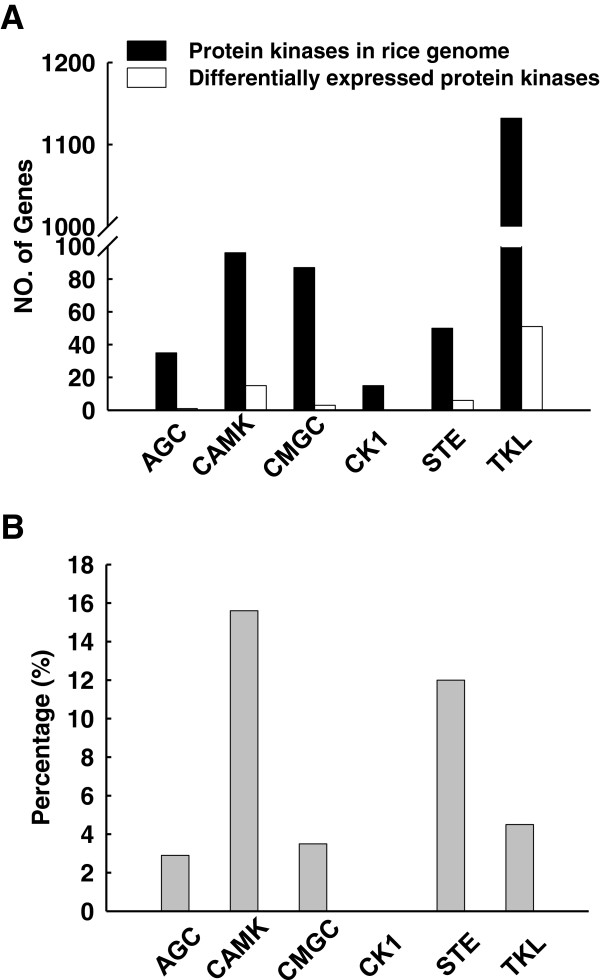
**Classification of protein kinase genes showing transcriptional changes in response to K**^**+**^**deficiency.** (**A**) Numbers of genes in different rice kinase groups showing transcriptional changes. (**B**) Percentages of genes showing transcriptional changes in different kinase groups.

According to the Rice Kinase Database [40] (http://phylomics.ucdavis.edu/kinase/index.shtml), there are 1,467 kinase genes corresponding to 1,934 transcripts in the rice genome. These protein kinases can be divided into six major phylogenetic groups; AGC, CAMK, GMGC, CK1, STE, and TKL [40]. In the present study, 123 kinase genes from five groups (all groups except CK1) were transcriptionally regulated by K^+^ deficiency. This represents 8.4% of the total kinase genes in rice. Among them, most changed kinase genes belong to TKL group (Figure 6A). TKL group mainly consists of receptor-like protein kinases. Many of them have been reported to play crucial roles in plant response to various external signals [41]. For example, the leucine-rich repeat (LRR) receptor-like protein kinases BRI1 and BAK1 are involved in brassinosteroid signal transduction in *Arabidopsis*[42,43]. In addition, RPK1 participates in ABA signaling pathway [44]. Therefore, we hypothesized that the transcriptionally changed genes in TKL group may be also involved in rice response to low-K^+^ stress. These proteins possibly sense external low-K^+^ signal and transduce it to downstream components via phosphorylation.

We also analyzed the percentages of kinase genes showing changes in transcription in each kinase group. A greater proportion of the kinases in the CAMK and STE groups (15.6% and 12%, respectively) showed changes in transcription compared with the other three groups (Figure 6B). This result indicated that kinases in these two groups may be more important in rice responses to K^+^ deficiency. In the CAMK group, the genes showing changes in transcription included ten *OsCIPK*s, two *OsSnRK1*-type, two *OsCPK*s, and one *OsCCaMK* (Table 2). In the STE group, four OsWNK protein genes and two MAP3K kinase genes showed transcriptional changes (Table 2). In our microarray data, many kinase genes from *OsCIPK* (CBL-interacting protein kinase) and *OsWNK* (with no K = lysine) families were transcriptionally regulated in rice seedlings subjected to K^+^ deficiency (Additional file 6, 7).

**Table 2:** **Genes encoding protein kinases and ion transporters showing changes in expression in rice subjected to K **^**+ **^**-deficiency **

**Groups**	**Gene Locus**	**Fold Change**			**Annotation**
		**6 h**	**3 d**	**5 d**	
CAMK kinase	Os08g0441100		2.39	1.82	OsCIPK6
	Os07g0150700		1.59	1.55	OsCIPK23
	Os03g0126800		1.42	1.54	OsCIPK9
	Os11g0113700			1.46	OsCIPK15
	Os12g0113500			1.46	OsCIPK14
	Os07g0678600	1.20	1.45	1.21	OsCIPK2
	Os02g0161000			1.28	OsCIPK26
	Os03g0319400		−1.33	−2.05	OsCIPK31
	Os07g0678300		−1.24	−1.72	OsCIPK29
	Os03g0339900		−1.21	1.27	OsCIPK10
	Os03g0289100		1.23		AMPKh.2-SnRK1-type kinase
	Os08g0484600		1.23		AMPKh.4-SnRK1-type kinase
	Os05g0489900	−1.26	−1.27	−1.71	OsCCaMK
	Os03g0688300		−1.48	−1.63	OsCPK9
	Os12g0169800			−1.31	OsCPK28
STE kinase	Os11g0114600	1.44	1.92	2.23	OsWNK7
	Os07g0584100		1.57	2.03	OsWNK5
	Os12g0114100	1.32	1.57	1.65	OsWNK8
	Os12g0162100		−1.27	−1.36	OsWNK9
	Os01g0699100		1.38	1.96	MAP3K.4
	Os05g0545400		1.67	1.60	MAP3K.19
K^+^ channels	Os05g0428700	1.20	1.78	1.65	K^+^ channel AKT2/3 – OsK3.1
	Os03g0752300		1.47	1.45	OsKCO1
	Os04g0445000		1.31	1.41	K^+^ channel SKOR - OsK5.1
	Os01g0648000	1.23			OsAKT1
HKT transporter	Os06g0701700	2.61	8.01	7.74	OsHKT2;1
HAK transporter	Os04g0401700	1.21	1.27	1.32	OsHAK1
	Os04g0613900		1.20	1.62	OsHAK11
	Os07g0669700		1.32	1.37	OsHAK7
	Os07g0679000		−1.23	1.22	OsHAK9
	Os06g0270200	−1.25	−1.41	−2.16	OsHAK24
	Os03g0337500		−1.24	−1.23	OsHAK8
	Os09g0376900			−1.21	OsHAK23
Pi transporter	Os01g0110100		1.51	2.06	OsPHO1;1
	Os04g0186400		2.29	3.35	OsPHT1;4
Nitrate transporter	Os02g0112600		−3.25	−6.10	A nitrate transporter
	Os02g0689900		−2.01	−3.16	Peptide transporter PTR2

CIPKs are plant-specific serine/threonine protein kinases that form protein complexes with calcium sensor CBLs (calcineurin B-like proteins) to perform functions [45,46]. A previous investigation in *Arabidopsis* showed that the protein kinase CIPK23 together with CBL1/9 activates AKT1-mediated K^+^ uptake in roots under low-K^+^ conditions [47,48]. Another recent study found that CIPK6 modulates the translocation of the K^+^ channel, AKT2, to the plasma membrane by interacting with CBL4, and enhances AKT2 activity at the plasma membrane [49]. Rice OsCIPKs show high similarity to *Arabidopsis* CIPKs in their amino acid sequence; therefore, similar regulatory mechanisms may also exist in rice. According to the sequences of *AtCIPKs* in *Arabidopsis*, Kolukisaoglu et al. (2004) identified at least 30 OsCIPKs from rice genome [50]. In NCBI and RGAP database, we can find 33 OsCIPKs in the updated annotations. In Affymetrix rice microarray, these 33 *OsCIPKs* genes are represented by 31 probe sets. Approximately one-third of *OsCIPKs* showed high expression levels in rice roots (average signal intensity values ≥ 500). In the present data, eight *OsCIPK* genes (*OsCIPK2, 6, 9, 10, 14/15, 23, 26*) were up-regulated and two *OsCIPK* genes (*OsCIPK29, 31*) were down-regulated under low-K^+^ stress (Table 2, Additional file 6). Increased expressions of these kinase genes may regulate activity of the K^+^ channel, enhancing K^+^ uptake under K^+^-deficient conditions.

WNK is another kind of serine/threonine protein kinase, which was first investigated in mammals [51]. The WNKs in mammals function in renal regulation of ion transport, especially in Na^+^ and K^+^ transport [39,52]. In higher plants, WNK homologs were identified in several plant species such as *Arabidopsis* (10 genes) and rice (9 genes) [53-55]. A recent report showed that AtWNK8 could phosphorylate the subunit of vacuolar H^+^-ATPase and regulate ion transport in *Arabidopsis*[56]. In rice, OsWNK1 is regulated by abiotic stress and plays a role in the internal circadian rhythm [55]. In our microarray experiments, five *OsWNKs* were strongly expressed in rice roots (average signal intensity values ≥ 500). In addition, *OsWNK5, 7, 8* were up-regulated and *OsWNK9* was down-regulated under low-K^+^ conditions (Table 2, Additional file 7).

Some kinases from these two families phosphorylate ion transporters and regulate ion transport in plant cells. It is likely that the expression levels of these kinase genes increased in rice seedlings under low-K^+^ conditions to regulate ion transporters, thereby enhancing transporter-mediated K^+^-uptake in rice roots.

### Membrane proteins and ion transporters

Uptake of K^+^ in plant cells is primarily mediated by K^+^ transporters and K^+^ channels located at the plasma membrane [57-59]. K^+^ transporters are major components of the high-affinity K^+^-uptake machinery that operates under low external K^+^ conditions [5,60,61]. Many high-affinity K^+^ transporter genes from different plant species are induced by low-K^+^ stress, such as *AtHAK5*, *HvHAK1*, and *OsHAK1*[8-12]. The increase in expression levels of genes encoding K^+^ transporters may be a rapid and direct strategy for plants to increase K^+^-uptake and overcome K^+^ deficiency. This appears to be a common regulatory mechanism among different plant species.

In the rice genome, the major K^+^ transporters are derived from *OsHAK* (27 genes) and *OsHKT* (9 genes) families [62,63]. In our microarray data, three *OsHAK* genes (*1*, *7* and *11*) were markedly up-regulated under low-K^+^ stress (Table 2, Additional file 8). This is consistent with a previous report that K^+^ starvation induced expression of *OsHAK1*[12]. We speculated that these K^+^ transporters may be involved in K^+^ uptake under low-K^+^ conditions.

For the *OsHKT* genes, all members of the *OsHKT* family were expressed at low levels in rice roots (Additional file 8). Only *OsHKT2;1* was strongly up-regulated during K^+^ deficiency (Table 2). This result is consistent with reports that homologs of *OsHKT2;1* in wheat and barley were significantly induced under low-K^+^ conditions [64,65]. It is interesting that this gene product was reported as a Na^+^ transporter, not a K^+^ transporter, when identified in heterologous expression systems [65,66]. In K^+^-starved rice roots, OsHKT2;1 increased the concentration of Na^+^ ions, so that these Na^+^ ions could serve as nutritional ions and enhance growth of rice seedlings under low-K^+^ conditions [67].

Besides K^+^ transporter genes, we found that the transcriptional levels of some phosphate and nitrate transporter genes changed in rice roots under K^+^-deficiency. In our microarray data, two phosphate transporter genes (*OsPHO1;1* and *OsPHT1;4*) were markedly up-regulated after 5 d of K^+^ starvation (Table 2). In contrast, two nitrate transporter genes (Os02g0112600 and Os02g0689900) were down-regulated at the same time (Table 2). A similar regulatory mechanism was also found in *Arabidopsis*. The expression levels of three *Arabidopsis* nitrate transporter genes (*AtNRT2;1*, *AtNRT2;3*, *AtNRT2;6*) were reduced under low-K^+^ conditions and quickly increased after K^+^ resupply [15]. In contrast, P- and N-deficiencies increased the expressions of K^+^ transporters such as *HAK5*, *KUP10*, *KUP11* in *Arabidopsis*[35,68]. These findings indicate that K, P, and N nutrient uptakes are inter-related. The deficiency of one type of nutrient ion may affect the absorption and translocation of other two types of ions in plants. This latent mechanism could facilitate the homeostasis of nutrient ions in plant cells. However, further research is required to clarify the details of this regulatory mechanism.

### Comparative analysis of low-K^+^ responses between rice and *Arabidopsis*

Rice and *Arabidopsis* are both model plants, the former being a model monocot and the latter a model dicot. It is meaningful to compare transcriptomic changes between these two species under low-K^+^ stress to understand which mechanisms are shared and which are specific to each plant, and to gain insight into common regulatory mechanisms. Previous investigations have indicated that many genes are transcriptionally regulated under P- and N-deficient conditions; this was observed in both *Arabidopsis*[26,27,68] and rice [18-21]. In contrast, fewer genes were induced by K^+^ deficiency in both *Arabidopsis*[9,15] and rice (present data; Additional file 9). These findings suggest that transcriptional regulation is probably more important in plant responses to low P and N stress than in the plant response to low-K^+^ stress.

To analyze transcriptomic differences between rice and *Arabidopsis* under low-K^+^ stress, we compared microarray data from *Arabidopsis*[9] with that in the present study. We reanalyzed their microarray data (four *Arabidopsis* ATH1 microarrays, two time points, no biological duplication) with the same data processing methods (1.2-fold cutoff threshold) used in this study. Then, the results of GO analysis from rice and *Arabidopsis* were compared (Additional file 10). Generally, the genes showing transcriptional changes in rice and *Arabidopsis* displayed similar GO distribution patterns in their responses to low-K^+^ stress. This result indicated that genes in the categories of metabolic process, membrane, and cation binding may play crucial roles in responses to low-K^+^ stress in both rice and *Arabidopsis*. However, there were also some differences. There was a greater proportion of genes related to the stress response and to development in *Arabidopsis* than in rice (Additional file 10), suggesting that these genes were more important in the response to low-K^+^ stress in *Arabidopsis*.

In present data, we found 33 hormone-related genes that respond to K^+^ deficiency in rice roots. Most of them (23) are related to auxin, including 10 *OsIAAs*, 4 Os*SUARs* and 2 *OsGHs* etc. These genes have been listed in “Additional file 11”. The transcriptional levels of several members from these gene families can be induced by auxin [69]. While, they are also up-regulated or down-regulated under low K^+^ conditions in rice roots, which indicates the link between auxin and K^+^ nutrient signaling. It has been reported that auxin is accumulated in central cylinder cells in the distal elongation zone of *Arabidopsis* roots after K^+^ deprivation [70]. Therefore it is hypothesized that auxin may also generate in rice roots after K^+^ deprivation and regulate rice root morphology under K^+^-deficient conditions. Armengaud et al., (2004) reported that many JA-related genes were induced after K^+^ deprivation, and highlighted a novel role of JA in *Arabidopsis* K^+^ nutrient signaling. However, it was noteworthy that these JA-related genes were mainly induced in *Arabidopsis* shoots, but less in roots. Therefore, it is suggested that different phytohormones may play roles in different organs to facilitate plant adapting K^+^-deficient stress.

These transcriptomic comparisons may provide some clues to understand differences between rice and *Arabidopsis* in their responses to low-K^+^ stress. However, further research is required to explore the differences in transcriptional regulation under nutrient deficiency between monocots and dicots.

## Conclusions

K^+^ deficiency is a common abiotic stress in the natural environment, and it inhibits plant growth and reduces crop production. In the present study, we analyzed Affymetrix microarrays to study the transcriptomic profiles of rice roots during the response to K^+^ deficiency. The microarray data showed that fewer genes are involved in the response to K^+^ deficiency than in the responses to P and N-deficiencies. Therefore, transcriptional regulation is probably more important in the plant response to low P- and low N-stress than to low-K^+^ stress. In total, 2,896 genes showed significant changes in transcription levels during low-K^+^ treatment. These were mainly in the GO categories of metabolic process, membrane, cation binding, kinase activity, transport, and so on. Many genes in protein kinase and ion transporter families were markedly up-regulated, suggesting that they may play important roles in rice responses to K^+^ deficiency. We also conducted a comparative analysis of transcriptomic changes between *Arabidopsis* and rice under low-K^+^ stress. The result indicated that monocots and dicots may share similar mechanisms in their responses to K^+^ deficiency, despite some differences. However, further research is required to clarify the differences in transcriptional regulation between monocots and dicots.

## Methods

### Plant growth condition and low-K^+^ stress treatment

Rice cultivar *Nipponbare* (*Oryza sativa* L. ssp. *japonica*) plants were cultured hydroponically in a growth chamber under the following conditions: relative humidity, 50–70%; 12-h light/12-h dark photoperiod; temperatures, 30°C days, 25°C nights.

The composition of the nutrient solution was as described by the International Rice Research Institute [23]. The solution contained 1.427 mM NH_4_NO_3_, 0.323 mM NaH_2_PO_4_·2H_2_O, 0.512 mM K_2_SO_4_, 0.998 mM CaCl_2_, 1.643 mM MgSO_4_·7H_2_O, 9.474 μM MnCl_2_·4H_2_O, 0.075 μM (NH_4_)_6_Mo_7_O_24_·4H_2_O, 18.882 μM H_3_BO_3_, 0.152 μM ZnSO_4_·7H_2_O, 0.155 μM CuSO_4_·5H_2_O, 0.031 mM FeSO_4_·7H_2_O, and 0.031 mM Na_2_EDTA·2H_2_O, (pH = 5.5). The nutrient solution was replaced every second day. First, the rice seedlings were cultured in normal solution for two weeks. Then, half of them were transferred to -K nutrient solution (lacking K_2_SO_4_) as the K^+^-deficient treatment (LK), and another half were transferred to normal solution (+K) as control (CK). Roots and shoots of both control and treated seedlings were collected at indicated times for further assays.

### Biomass and K^+^ content measurement

The roots and shoots of control and treated rice seedlings were collected at various times during the K^+^-deficiency treatment (6 h, and 1, 3, 5, and 7 d). The root and shoot tissues were harvested separately, dried at 80°C for 48 h, and then weighed (dry weight). To measure K^+^ content, dry samples were incinerated in a muffle furnace at 575°C for 5 h, the residue was dissolved in 0.1 N HCl, and the K^+^ content was measured by atomic absorption spectrophotometry (Hitachi Z-5000). Three biological replicates were used for phenotypic tests, biomass and K^+^ content measurements. The *t* - test (* *P* < 0.05, ** *P* < 0.01) was used to analyze the statistical significance.

### RNA isolation, target preparation, and hybridization to Affymetrix genechips

Fresh roots of both control and treated rice seedlings were collected at indicated times during the K^+^-deficiency treatment (6 h, 3 and 5 d). The roots were immediately frozen in liquid nitrogen and stored at −80°C until further RNA extraction. Every RNA sample was derived from five independent seedlings. Total RNA was extracted from rice roots using Trizol reagent (Invitrogen, Carlsbad, CA, USA). Quality characterization of RNA samples was determined and confirmed using a NanoDrop 2000 fluorospectrometer and formaldehyde denaturing gel electrophoresis. Then, the total RNA was purified using the RNeasy Mini kit (Qiagen, Valencia, CA, USA). The purified RNA was used to generate first-strand cDNA in a reverse transcription reaction containing One-Cycle Target Labeling and Control reagents (Affymetrix, Santa Clara, CA, USA) at 42°C for 2 h and 16°C for 1 h. The double-stranded cDNAs were used to generate cRNA via an in vitro transcriptional reaction. The cRNA was labeled with biotin, and 20 μg labeled cRNA was fragmented. The size distribution of cRNAs and fragmented cRNAs was assessed using an Eppendorf Biophotometer and electrophoresis. Fragmented cRNA (15 μg) was added to 300 μL hybridization solution, and then 200 μL of this mixture was used for hybridization on Affymetrix rice genome arrays for 16 h at 45°C. The standard wash and double-stain protocols were applied using a fluidics station (Affymetrix GeneChip Fluidics Station 450). The arrays were scanned on a genechip scanner (Affymetrix GeneChip Scanner 3000).

### Microarray data processing and analysis

First, the scanned arrays were analyzed using Affymetrix GCOS 1.0 (MAS 5.0) software to generate raw data, which were saved as CEL files. Then, DNA-Chip Analyzer (dChip) 2007 software [25] (http://www.dchip.org) was used to normalize all 18 array sets. A gene was considered to be expressed when it had detection call “P” in all three replicates, and when its detection *P*-value was less than 0.05. We used GeneSpring GX 11 software to analyze the correlation coefficients between every pair of arrays. The genes showing changes in transcription in response to K^+^ deficiency were analyzed using dChip 2007 software. A gene was considered to show transcriptional changes when it satisfied two conditions: (1) up-regulation or down-regulation more than 1.2-fold after K^+^-deficiency treatment; (2) a fold-change *P-*value of less than 0.05. A negative or positive value represents downward or upward regulation, respectively. We conducted hierarchical cluster analysis of genes showing transcriptional changes using TIGR MeV 4.2 software [71] (http://www.tm4.org). The genes up- or down-regulated at least at one time point were collected respectively, which were used for Venn diagram analysis. The AgriGO web service (http://bioinfo.cau.edu.cn/agriGO/analysis.php) was used to analyze the functional categories of genes showing transcriptional changes. Microsoft Excel and Access were used to extract and manage microarray data.

### Real-time PCR analysis

The RNA samples for microarray experiment (three biological replicates) were also used for real-time PCR assays in order to ensure the reliability and repeatability of the results. To eliminate genomic DNA contamination, total RNA was treated with DNase I (RNase Free) (Takara, Dalian, China). Then, the total RNA was used to synthesize cDNA in a reverse transcription reaction using random primers (Promega, Madison, WI, USA). The cDNA samples were diluted to 4 ng/μL. Three biological replicates were performed using the Power SYBR Green PCR Master Mix (Applied Biosystems, Foster City, CA, USA) on a 7500 Real Time PCR System machine (Applied Biosystems) according to the manufacturer’s protocols. The gene-specific primers (Additional file 12) were designed using Primer 3 (http://frodo.wi.mit.edu/primer3/input.htm) and DNAMAN software. Amplification of *18S rRNA* was used as an internal control to normalize the data.

## Competing interests

The authors declare that they have no competing interests.

## Authors’ contributions

WY conceived the study. MTL carried out phenotypic tests and physiological analyses. MTL carried out RNA extractions, performed real-time RT-PCR analyses, and drafted the manuscript. WY and MTL designed the experiments and analyzed microarray data. WY and WWH supervised the project and finalized the paper. All authors read and approved the final manuscript.

## Supplementary Material

Additional file 1 Correlation coefficient analysis of 18 microarrays. Click here for file

Additional file 2 **Numbers of probe sets under control (CK) and low-K**^**+**^**(LK) conditions.** Click here for file

Additional file 3 **All genes showing transcriptional changes during K**^**+**^**-deficiency.** Click here for file

Additional file 4 **Functional classification of genes showing transcriptional changes (up-regulated and down-regulated) at 6 h (A), 3 d (B), and 5 d (C) of K**^**+**^**deficiency.** AgriGO web-based tool was used to analyze GO categories of differentially expressed genes. Numbers of genes showing transcriptional changes in 13 main biological categories are shown. Click here for file

Additional file 5 **Genes encoding calcium sensors and peroxidase proteins showing transcriptional changes during K**^**+**^**-deficiency treatment.** Click here for file

Additional file 6 **Expression levels of*OsCIPK*genes during K**^**+**^**deficiency.** Click here for file

Additional file 7 **Expression levels of*OsWNK*genes during K**^**+**^**deficiency.** Click here for file

Additional file 8 **Expression levels of*OsHAK*(A) and*OsHKT*(B) genes during K**^**+**^**deficiency.** Click here for file

Additional file 9 **Comparison of differentially expressed genes between rice and*Arabidopsis*in responses to K**^**+**^**deficiency.** Click here for file

Additional file 10 **Comparison of GO classifications between rice and*Arabidopsis*in responses to K**^**+**^**deficiency.** Click here for file

Additional file 11 **Hormone- and auxin-responsive genes showing transcriptional changes during K**^**+**^**deficiency.** Click here for file

Additional file 12 Gene-specific primers used in real-time PCR experiments. Click here for file
