# Supplementary material for: Transcriptome analysis of rice root responses to potassium deficiency
Source: BMC Plant Biol. 2012 Sep 10;12:161. doi: 10.1186/1471-2229-12-161 (PMC3489729; doi:10.1186/1471-2229-12-161)
Supplement: Additional file 1 — Correlation coefficient analysis of 18 microarrays. [file 1471-2229-12-161-S1.pdf]

Array Name	CK-6h-1	CK-6h-2	CK-6h-3	LK-6h-1	LK-6h-2	LK-6h-3	CK-3d-1	CK-3d-2	CK-3d-3	LK-3d-1	LK-3d-2	LK-3d-3	CK-5d-1	CK-5d-2	CK-5d-3	LK-5d-1	LK-5d-2	LK-5d-3
**CK-6h-1**	1.0000																	
**CK-6h-2**	0.9933	1.0000																
**CK-6h-3**	0.9933	0.9977	1.0000															
**LK-6h-1**	0.9957	0.9954	0.9953	1.0000														
**LK-6h-2**	0.9887	0.9950	0.9942	0.9915	1.0000													
**LK-6h-3**	0.9901	0.9970	0.9958	0.9948	0.9963	1.0000												
**CK-3d-1**	0.9885	0.9857	0.9870	0.9867	0.9826	0.9838	1.0000											
**CK-3d-2**	0.9874	0.9902	0.9901	0.9872	0.9896	0.9892	0.9955	1.0000										
**CK-3d-3**	0.9842	0.9892	0.9890	0.9847	0.9886	0.9893	0.9932	0.9982	1.0000									
**LK-3d-1**	0.9780	0.9785	0.9782	0.9793	0.9763	0.9793	0.9882	0.9884	0.9877	1.0000								
**LK-3d-2**	0.9727	0.9807	0.9801	0.9771	0.9791	0.9828	0.9840	0.9880	0.9889	0.9949	1.0000							
**LK-3d-3**	0.9726	0.9777	0.9783	0.9745	0.9802	0.9805	0.9847	0.9896	0.9897	0.9944	0.9962	1.0000						
**CK-5d-1**	0.9875	0.9889	0.9880	0.9884	0.9856	0.9873	0.9923	0.9941	0.9921	0.9863	0.9850	0.9826	1.0000					
**CK-5d-2**	0.9845	0.9897	0.9878	0.9857	0.9890	0.9888	0.9889	0.9939	0.9927	0.9831	0.9848	0.9822	0.9967	1.0000				
**CK-5d-3**	0.9844	0.9877	0.9885	0.9842	0.9889	0.9873	0.9919	0.9958	0.9948	0.9839	0.9852	0.9870	0.9947	0.9963	1.0000			
**LK-5d-1**	0.9722	0.9730	0.9727	0.9735	0.9727	0.9733	0.9801	0.9818	0.9802	0.9911	0.9906	0.9899	0.9835	0.9818	0.9835	1.0000		
**LK-5d-2**	0.9703	0.9764	0.9750	0.9732	0.9753	0.9779	0.9780	0.9820	0.9817	0.9900	0.9933	0.9907	0.9836	0.9847	0.9843	0.9965	1.0000	
**LK-5d-3**	0.9628	0.9730	0.9706	0.9663	0.9749	0.9753	0.9719	0.9792	0.9797	0.9851	0.9924	0.9903	0.9789	0.9814	0.9807	0.9924	0.9965	1.0000
