# Supplementary material for: Transcriptome analysis of rice root responses to potassium deficiency
Source: BMC Plant Biol. 2012 Sep 10;12:161. doi: 10.1186/1471-2229-12-161 (PMC3489729; doi:10.1186/1471-2229-12-161)
Supplement: Additional file 2 — Numbers of probe sets under control (CK) and low-K+(LK) conditions. [file 1471-2229-12-161-S2.pdf]

Probe sets expressed in	No.of Probe sets	Percentage in rice array (57,194 probe sets)
CK-6 h	24,768	43.3
LK-6 h	25,403	44.4
CK-3 d	24,192	42.3
LK-3 d	24,508	42.9
CK-5 d	25,051	43.8
LK-5 d	25,392	44.4
