# Supplementary figures and images for: Transcriptome analysis of rice root responses to potassium deficiency
Source: BMC Plant Biol. 2012 Sep 10;12:161. doi: 10.1186/1471-2229-12-161 (PMC3489729; doi:10.1186/1471-2229-12-161)

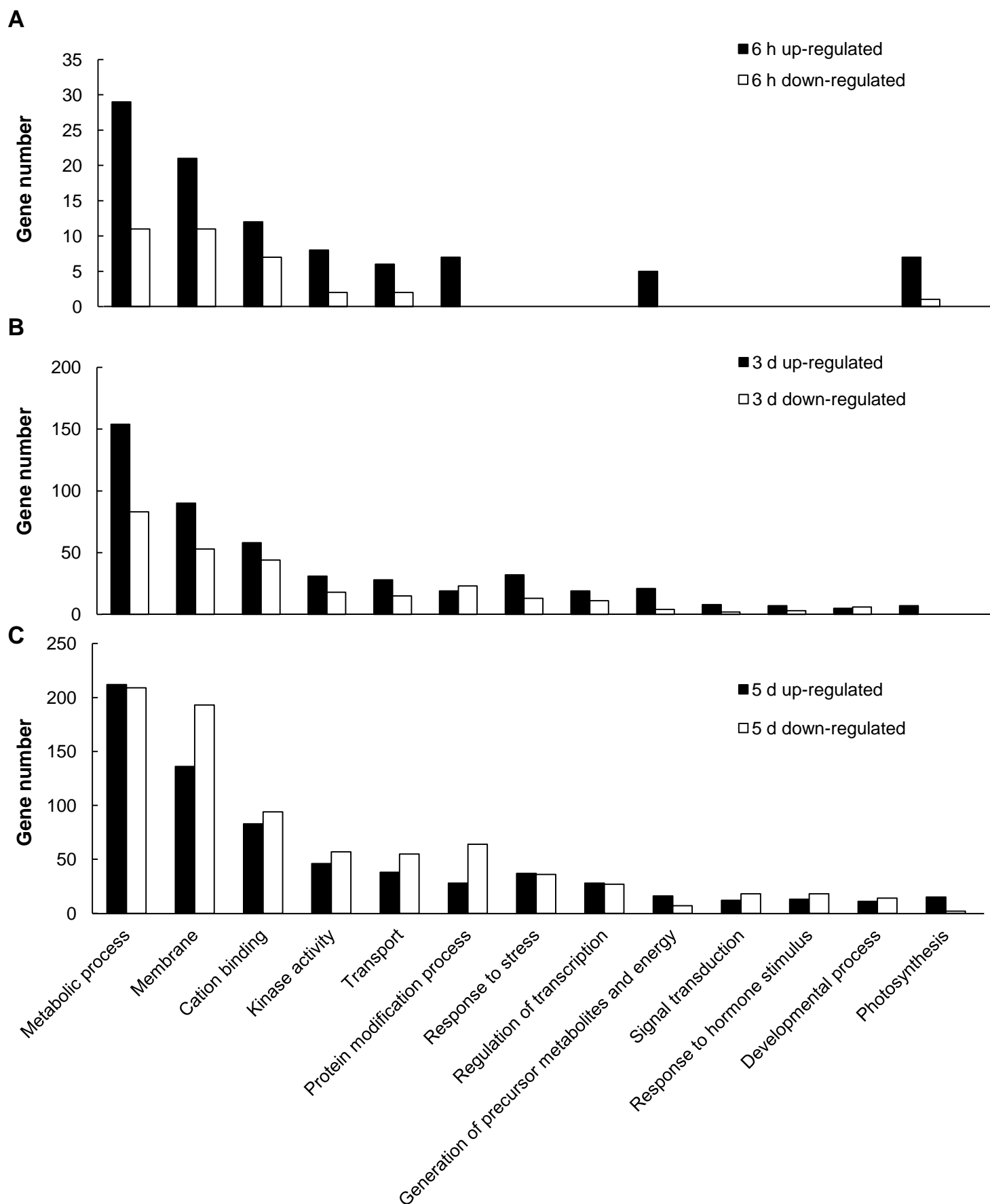

Supplement: Additional file 4 — Functional classification of genes showing transcriptional changes (up-regulated and down-regulated) at 6 h (A), 3 d (B), and 5 d (C) of K+deficiency. AgriGO web-based tool was used to analyze GO categories of differentially expressed genes. Numbers of genes showing transcriptional changes in 13 main biological categories are shown. [file 1471-2229-12-161-S4.pdf]

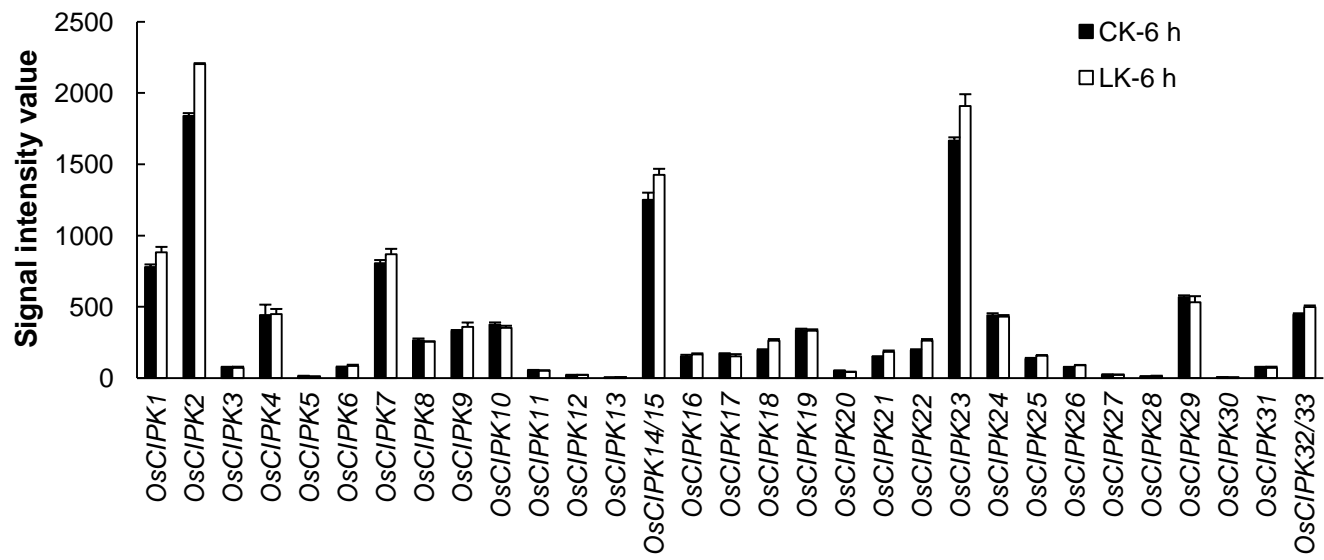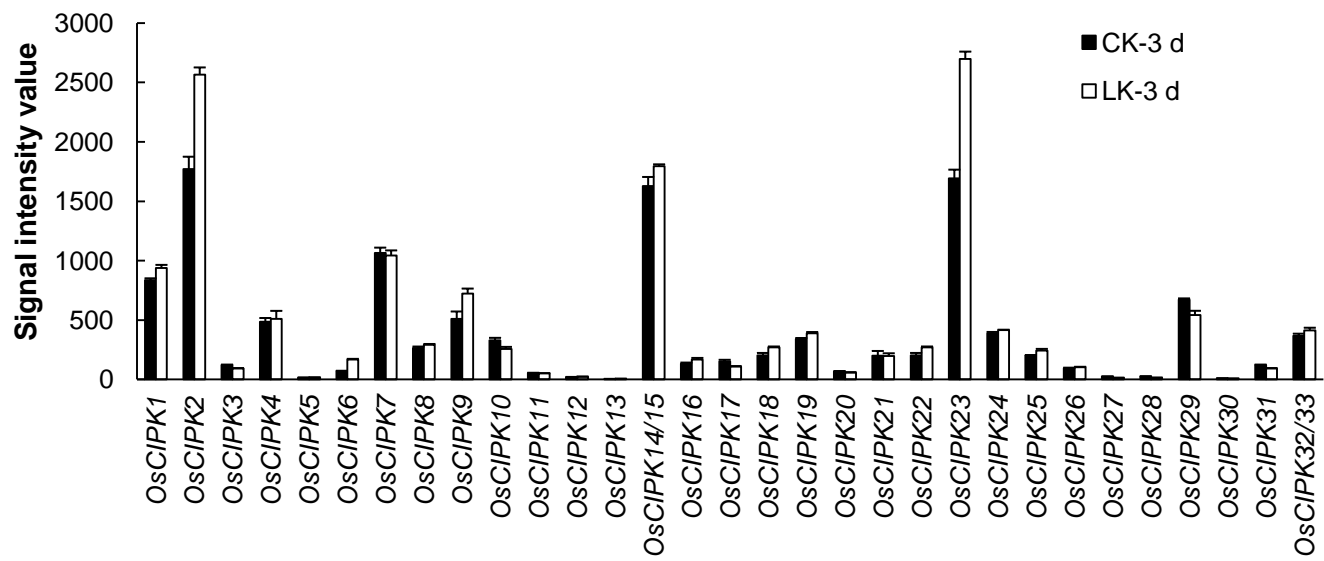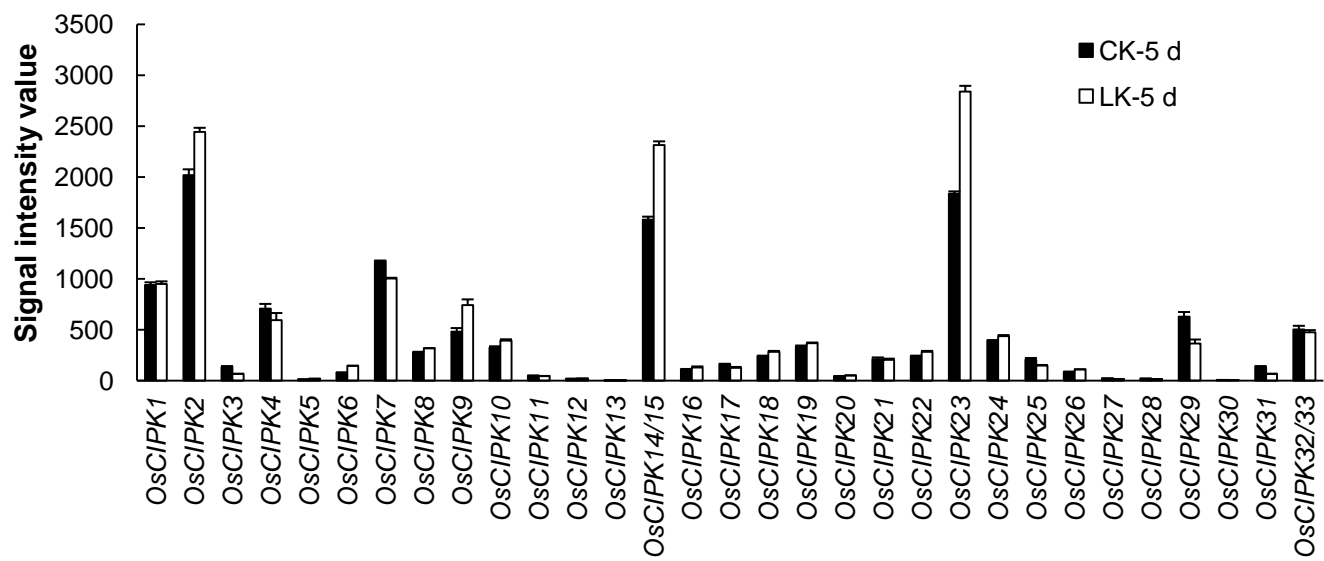

Supplement: Additional file 6 — Expression levels ofOsCIPKgenes during K+deficiency. [file 1471-2229-12-161-S6.pdf]

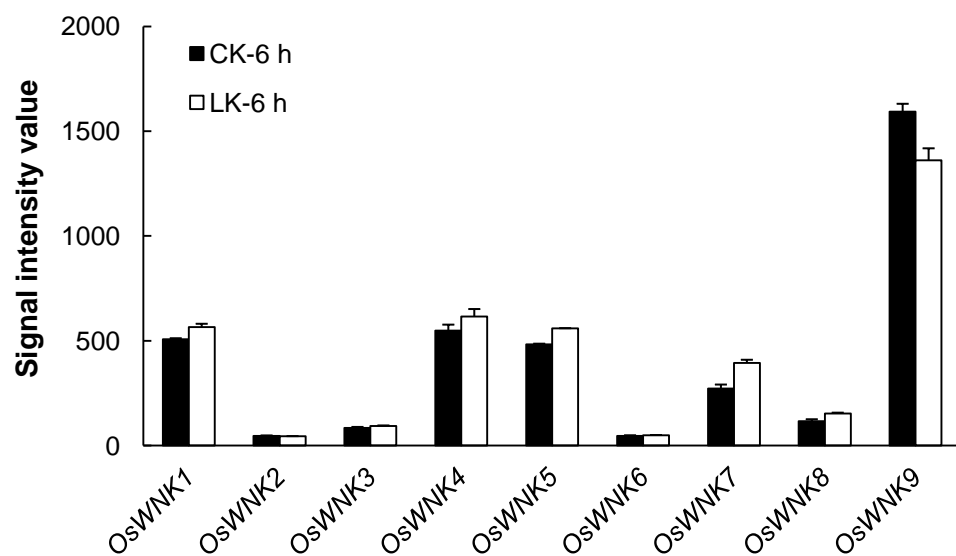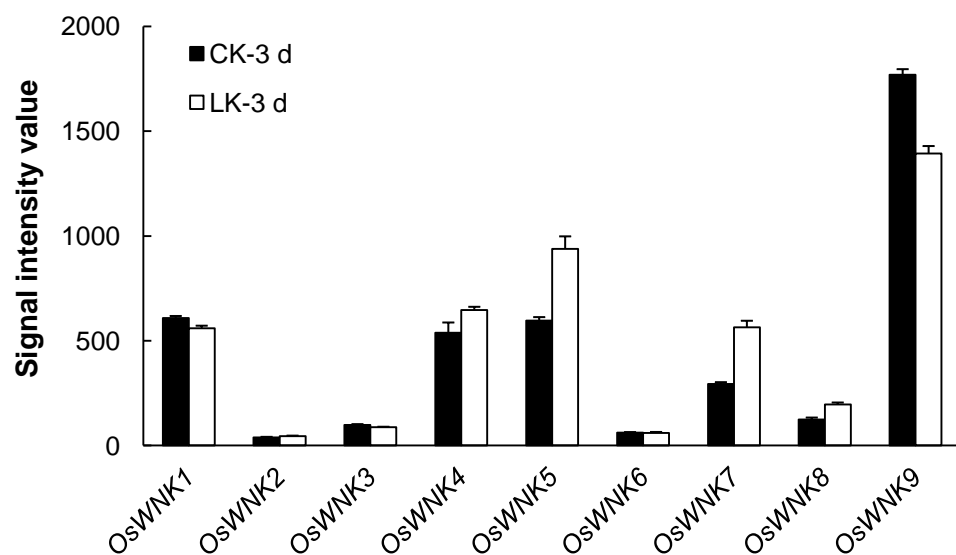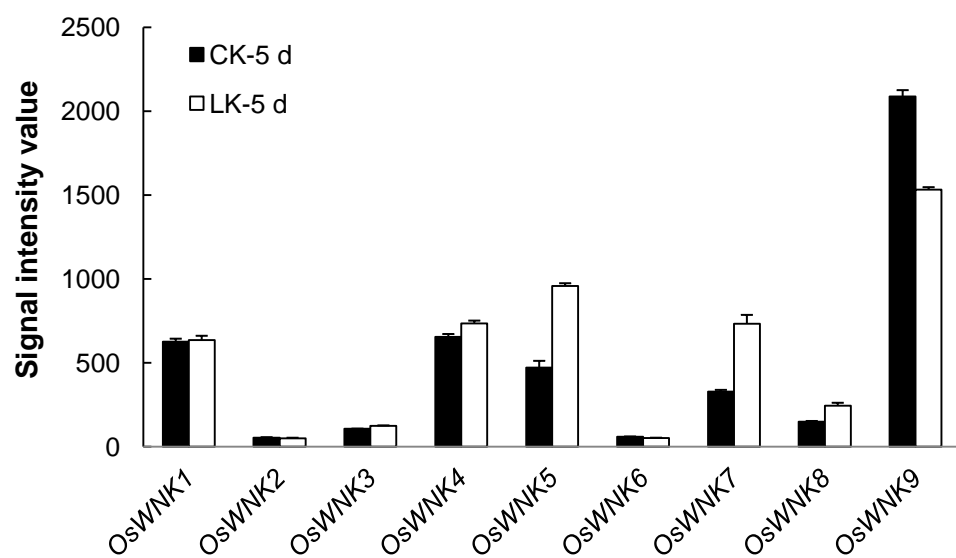

Supplement: Additional file 7 — Expression levels ofOsWNKgenes during K+deficiency. [file 1471-2229-12-161-S7.pdf]

**A**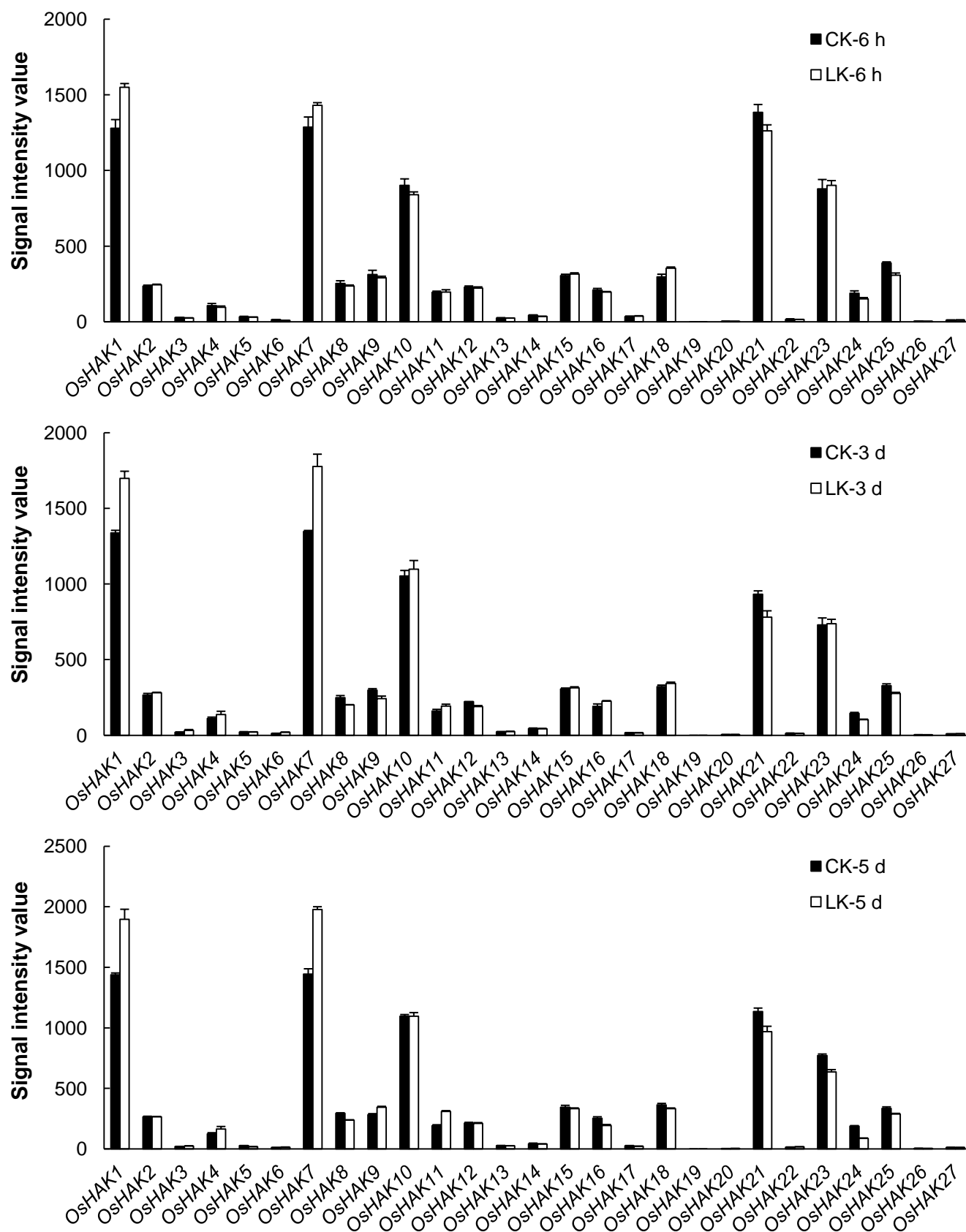

**B**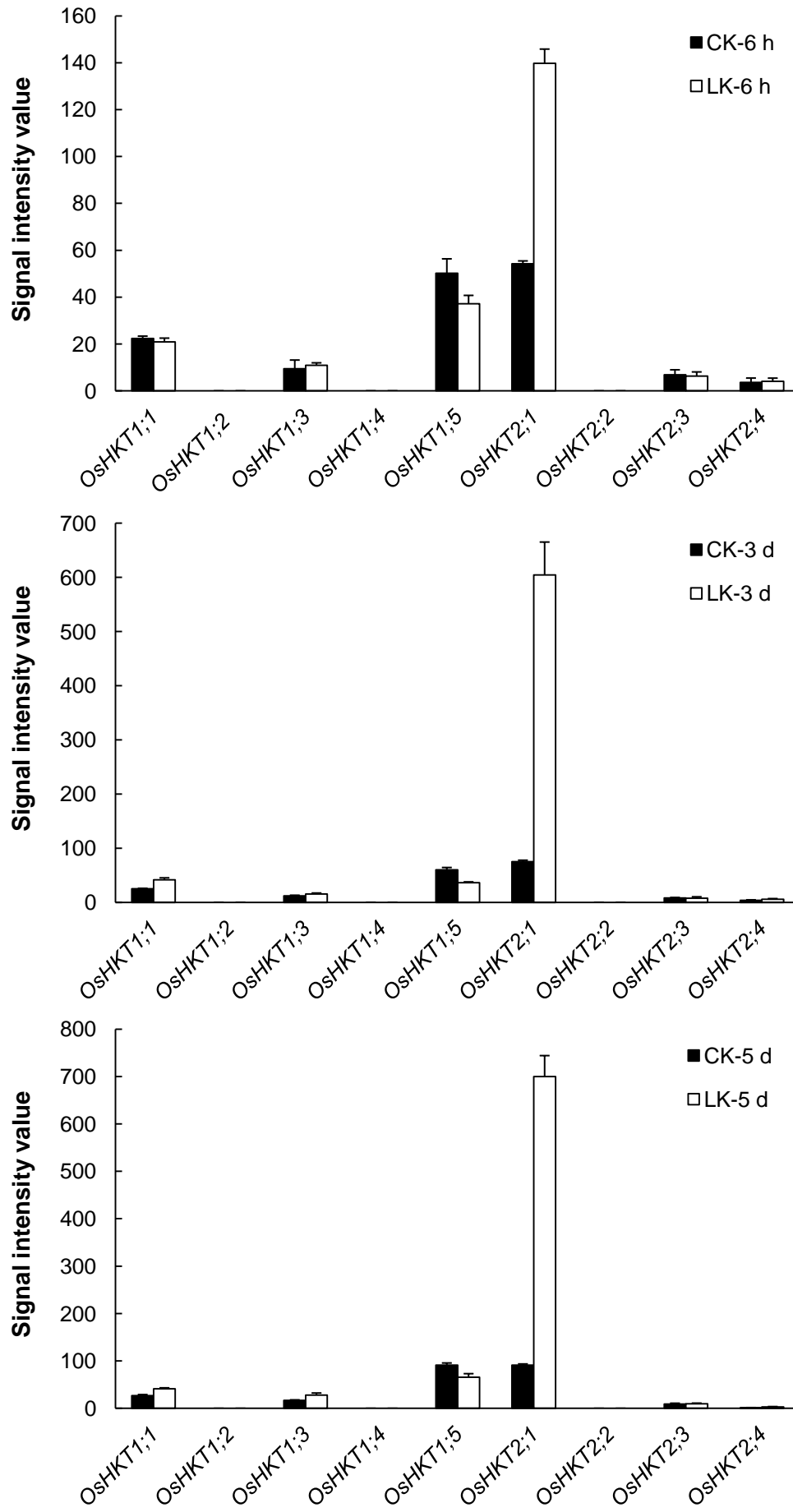

Supplement: Additional file 8 — Expression levels ofOsHAK(A) andOsHKT(B) genes during K+deficiency. [file 1471-2229-12-161-S8.pdf]

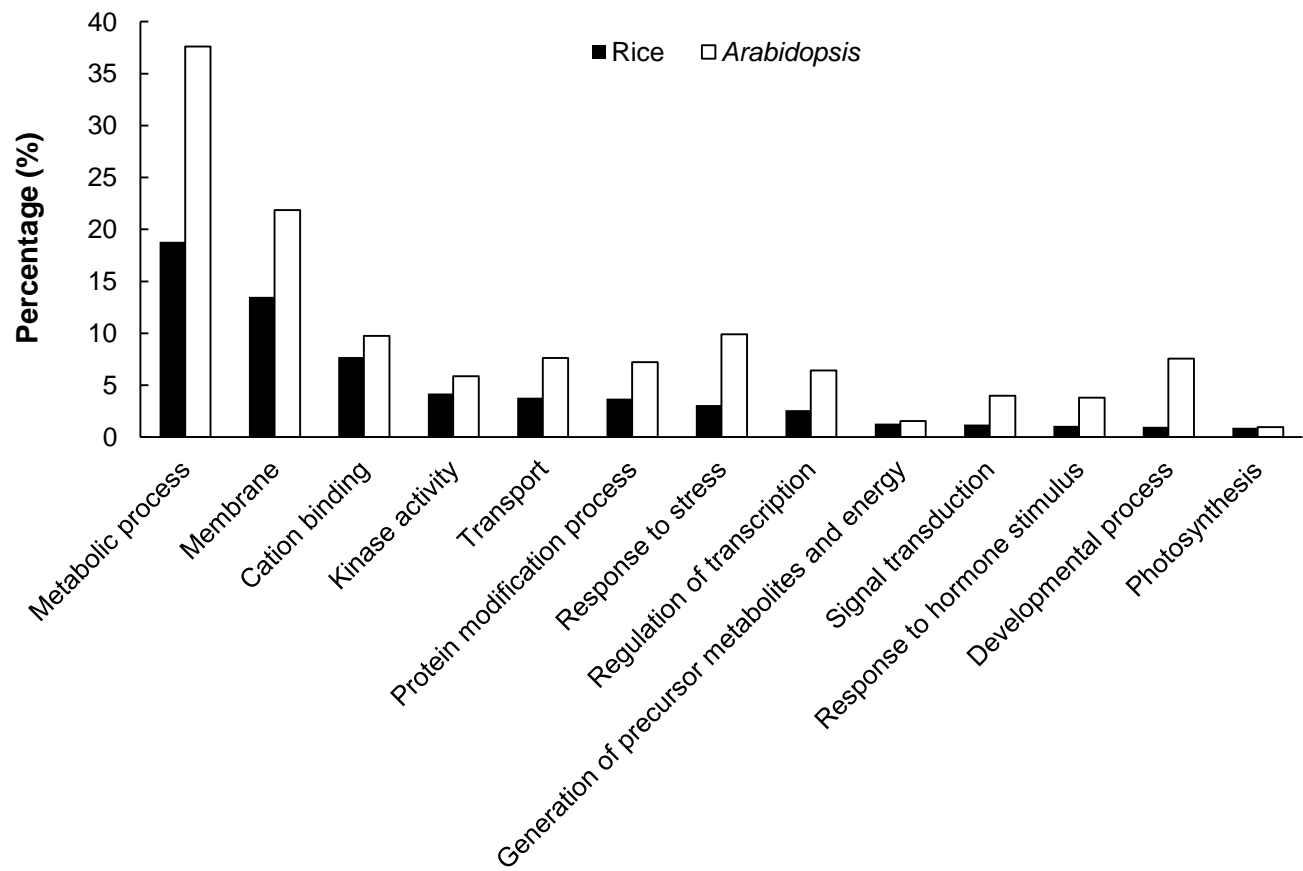

Supplement: Additional file 10 — Comparison of GO classifications between rice andArabidopsisin responses to K+deficiency. [file 1471-2229-12-161-S10.pdf]
