# Supplementary material for: Transcriptome analysis of rice root responses to potassium deficiency
Source: BMC Plant Biol. 2012 Sep 10;12:161. doi: 10.1186/1471-2229-12-161 (PMC3489729; doi:10.1186/1471-2229-12-161)
Supplement: Additional file 9 — Comparison of differentially expressed genes between rice andArabidopsisin responses to K+deficiency. [file 1471-2229-12-161-S9.pdf]

Analysis methods	Culture methods	No. of genes	Percentage in genome	Reference
Rank product statistics and iterative group analysis (FDR% < 1), Roots, n = 3	*Arabidopsis* seedlings grown on K^+-free medium in petri dishes for two weeks after germination.	299	1.25	Armengaud et al., 2004
Fold Change ≥ 2 and *P*-value ≤ 0.04, Roots, n = 1	*Arabidopsis* root response to low K^+ stress in the treatment time points of 2 d and 4 d under K^+-free hydroponic medium.	391	1.63	Gierth et al., 2005
Fold Change ≥ 2 and *P*-value ≤ 0.05, Roots, n = 3	Rice root response to low K^+ stress in the treatment time points of 6 h, 3 d and 5 d under K^+-free hydroponic medium.	356	0.71	Present data
