# Supplementary material for: Transcriptome analysis of rice root responses to potassium deficiency
Source: BMC Plant Biol. 2012 Sep 10;12:161. doi: 10.1186/1471-2229-12-161 (PMC3489729; doi:10.1186/1471-2229-12-161)
Supplement: Additional file 12 — Gene-specific primers used in real-time PCR experiments. [file 1471-2229-12-161-S12.pdf]

Probe ID (Gene ID)	Forword and reverse primers	Aonotation
Os.2617.2.S1_a_at (Os06g0701700)	5′-TCTGACACTTTCCAGCCTCA-3′ 5′-TGCTTGCCGAGTTGATTGTA-3′	OsHKT2;1
Os.23476.1.A1_at (Os01g0598200)	5′-TTGACCAGGCTGACACAAAA-3′ 5′-GCAACACCAGGTTTCAGGTT-3′	OsCBL5
Os.55509.1.S1_at (Os08g0441100)	5′-CAACATCGTCACCCTGTACC-3′ 5′-CGGAGGAAGCTCTATGCTCA-3′	OsCIPK6
Os.56004.1.S1_at (Os03g0347500)	5′-TGAATCGCATGCATCTTAGC-3′ 5′-CACGTGTCCCCAATCATACA-3′	OsSWEET12
Os.53403.1.S1_s_at (Os08g0508700)	5′-CGTTTGATGGATTGCCTTTT-3′ 5′-CGCCCTTCTCAAGTTCTCAG-3′	Ethylene-insensitive 3, putative, expressed
Os.53584.1.S1_at (Os02g0237100)	5′-TGGGTCATGGCATCAGATTA-3′ 5′-CTTGCATCATCCTCGGTGTA-3′	Spermidine synthase, putative, expressed
Os.2245.1.S1_at (Os05g0506000)	5′-CGAGCTAAGCAGCTTCTCCA-3′ 5′-AACAAGCAATTTGGTCCCTTA-3′	Tetratricopeptide repeat, domain containing protein
Os.9739.1.S1_at (Os04g0142400)	5′-GCCTCGATGAGGAAAACAAG-3′ 5′-CACATTACCGTGGTGACGAA-3′	Expressed protein
Os.47802.1.A1_at (Os12g0555000)	5′-ATTCATCGCGTGCATGATAA-3′ 5′-AAATGATCTCACCAACACTGAAAA-3′	Pathogenesis-related Bet v I family protein, putative, expressed

Os.5242.1.S1_at<br>(Os01g0795000)	5′-GCATAACCTGGGCAAAACAT-3′<br>5′-AATTGACCATCCATCGAAGTG-3′	OsSub6-Putative Subtilisin homologue, expressed
OsAffx.24308.1.S1_s_at<br>(Os02g0286933)	5′-GTTTGAGTGCTTGGTTCCCT-3′<br>5′-AGAAGAGATGGGTGAGGCAG-3′	Uncharacterized cys-rich domain containing protein, putative, expressed
OsAffx.28867.2.S1_at<br>(Os07g0611500)	5′-CAACTGATGCCCACATAACACA-3′<br>5′-GGCGAAGAAGGAGATGGTCT-3′	DC1 domain-containing protein, putative, expressed
